# Value of Ultrasonography in Detection of Diaphragmatic Injuries Following Thoracoabdominal Penetrating Trauma; a Diagnostic Accuracy Study

**Published:** 2019-08-18

**Authors:** Ali Sharifi, Amir Kasraianfard, Abdolhamid Chavoshi Khamneh, Soheila Kanani, Mohamedali Aldarraji, Mohammad Ali Seif-Rabiei, Amir Derakhshanfar

**Affiliations:** 1 Department of General Surgery, Hamadan University of Medical Sciences, Hamadan, Iran.; 2 Department of General Surgery, Iran University of Medical Sciences, Tehran, Iran.; 3 Department of Emergency Medicine, Hamadan University of Medical Sciences, Hamadan, Iran.; 4 General physician, Shahed Medical faculty, Tehran, Iran.; 5 Department of Social Medicine, Hamadan University of Medical Sciences, Hamadan, Iran.

**Keywords:** Wounds, stab, diaphragm, wounds and injuries, focused assessment with sonography of trauma, laparoscopy

## Abstract

**Introduction:**

Diagnosis of diaphragmatic rupture following thoracoabdominal penetrating trauma is very challenging in asymptomatic patients with stable vital signs. This study aimed to evaluate the diagnostic accuracy of focused assessment with sonography for trauma (FAST) in this regard.

**Methods:**

This cross-sectional study was performed on patients referring to emergency department due to left thoracoabdominal stab wound during 2 years. All patients initially underwent ultrasonography and the screening performance characteristics of FAST in detection of diaphragmatic injuries were calculated, considering the findings of diagnostic laparoscopy as the gold standard test.

**Results:**

Twenty-four patients with the mean age of 33 ± 10.64 years (16-61 years) were studied (100% male). The mean chest wall laceration size was 2.7 ± 2.7 cm (1-10 cm) and the most frequent location of wounds was posterior chest wall (42%) and in the 6^th^ and 7^th ^intercostal space (50%). Diaphragmatic rupture was seen in 4 (16.7%) patients based on diagnostic laparoscopy. Sensitivity, specificity, positive and negative predictive values, and positive and negative likelihood ratio of FAST in detection of diaphragmatic raptures were 50% (95% CI: 9.18 – 90.8), 100% (95% CI: 79.9 – 100.0), 100% (95% CI: 19.8 – 100.0), 9.1% (95% CI: 1.5 – 30.6), Infinity, and 0.1 (95% CI: 0.02 – 0.37), respectively. The overall accuracy of FAST in this regard was 75.0% (95% CI: 42.3 – 100.0).

**Conclusion:**

In patients with penetrating trauma to the left thoracoabdominal region, FAST cannot be the definitive alternative to diagnostic laparoscopy to detect diaphragm rupture.

## Introduction

Thoracoabdominal penetrating trauma is one of the common problems in surgical emergency centres. In patients with a penetrating trauma and unstable hemodynamic status, the primary and standard approach to diagnosis and treatment is surgical intervention, which can be explorative laparotomy or thoracotomy based on clinical assessment (1). Diagnosis of diaphragmatic rupture in patients with thoracoabdominal penetrating trauma is controversial in those who are hemodynamically stable and asymptomatic (2). In some clinical centres, those with penetrating lacerations of the thoracoabdominal area routinely undergo surgical explorations (3). Using this method, all diaphragmatic ruptures are detected, but a large number of patients undergo negative laparotomy, which leads to complications such as post-surgical adhesion or surgery wound site infections. 

In addition, this method is very time consuming and costly, especially in the crowded trauma centres with a large number of visitors. Therefore, several diagnostic methods including chest radiography, computed tomography (CT) scan and diagnostic peritoneal lavage have been used to diagnose diaphragmatic rupture, but their diagnostic accuracies have been reported as very poor in various studies (4).

Focused assessment with sonography for trauma (FAST) is a very suitable diagnostic method for patients with trauma, which is very easy to use and does not require general anaesthesia and is not likely to cause any harm to the patient (5-7). Using ultrasound has a sensitivity of 94%, specificity of 98% and approximate accuracy of 95% for detecting intra-abdominal bleeding and visceral injury (8). In this study, we aimed to determine the accuracy of FAST in detecting diaphragmatic rupture in patients with stab wound of sharp objects in thoracoabdominal region and to compare the results with diagnostic laparoscopy.

## Methods

***Study design and setting***

This was a cross-sectional (diagnostic accuracy) study conducted on all patients who were referred to the emergency department of Besat Hospital, Hamadan, Iran, following thoracoabdominal penetrating trauma, from 2016 to 2018. The screening performance characteristics of FAST sonography in detection of diaphragmatic injuries were calculated, considering the findings of diagnostic laparoscopy as the gold standard test. The study protocol was approved by the Ethics committee of Hamadan University of Medical Sciences (Ethics code: IR.UMSHA.REC.1397.169).

**Table 1:** Baseline characteristics of the studied patients

**Variables**	**Total **	**Ruptured diaphragm**		**P value **
		**Yes (n = 4)**	**No (n = 20)**	
**Age (year)**				
Mean ± SD	33 ± 10.64	31.2 ± 5.4	33.3 ± 11.5	0.91
**Time to reach the hospital (minute)**				
Mean ± SD	38.5 ± 16	38.2 ± 16.3	40 ± 16.8	0.73
**Chest wall laceration length (cm)**				
Mean ± SD	2.7 ± 2.2	2.9 ± 2.3	1.6 ± 0.5	0.24
**Hemothorax volume (ml)**				
Mean ± SD	125 ± 134	74 ± 70	380 ± 57	> 0.001
Location of wound (In thorax) *				
Posterior	10 (41.7)	0 (0.0)	10 (50.0)	0.009
Anterior	8 (33.3)	1 (25.0)	7 (35.0)	
Maxillary	6 (25.0)	3 (75.0)	3 (15.0)	
Location of wound (in intercostal space) *				
5^th^	3 (12.5)	0 (0.0)	3 (15)	0.013
6^th^	6 (25)	0 (0.0)	6 (30)	
7^th^	6 (25)	1 (25)	5 (25)	
8^th^	3 (12.5)	2 (50)	1 (5)	
9^th^	2 (8)	1 (25)	1 (5)	
10^th^	2 (8)	0 (0.0)	2 (10)	
11^th^	2 (8)	0 (0.0)	2 (10)	
Chest tube drainage*				
Hemothorax	11 (45.8)	0(0.0)	11 (55.0)	0.002
Pneumothorax	7 (29.2)	0 (0.0)	7 (35.0)	
Hemo-pneumothorax	6 (25.0)	4 (100.0)	2 (10.0)	

*Data are reported as number (%).

**Figure 1:**
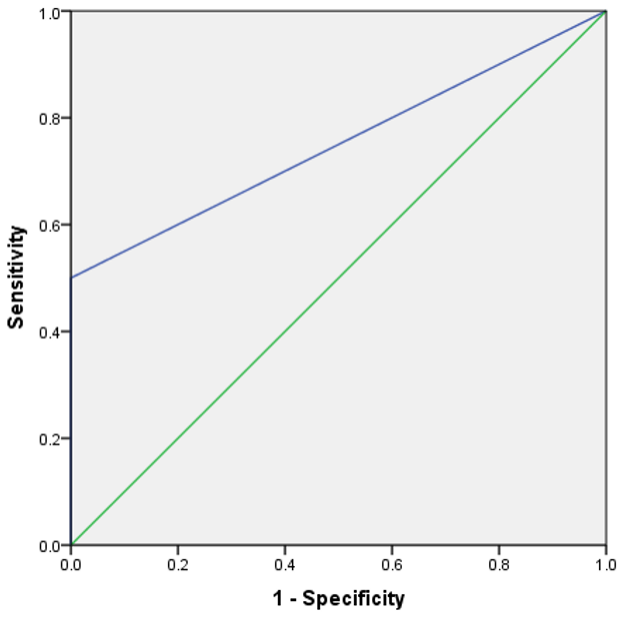
Area under the receiver operating characteristic (ROC) curve of ultrasonography in detection of diaphragmatic injuries following penetrating trauma

A written informed consent was obtained from patients. The research team was aware of all the provisions of the Helsinki declaration on the Ethics of medical research including human cases, and fully committed to its observance.

***Participants***

All patients referring to the emergency department of the mentioned hospital following injury by a sharp object to the thoracoabdominal area in the upper boundary of the fourth intercostal space (anterior), the sixth intercostal space (lateral), the eighth intercostal space (posterior) and the lower edge of the ribs, during two years of study, were enrolled. 

Patients who presented with unstable vital signs, had previous history of unrepaired diaphragmatic rupture, were stabbed with a sharp object located outside the thoracoabdominal area, had no evidence of hemothorax or pneumothorax in chest CT scan, had laparoscopy contraindications including history of obstructive pulmonary and cardiovascular diseases, and had generalized peritonitis were excluded.

***Study protocol***

All patients underwent chest CT scan on admission to the emergency department as a triage tool for penetrating thoracoabdominal trauma. Patients with negative CT scan were excluded from further investigation. In the presence of hemothorax or pneumothorax in the CT scan, patients were subjected to FAST, in which 3 spaces inside the abdomen including Morrison space (between the liver and the right kidney), splenorenal space (between the spleen and the left kidney) and the pelvis were examined for the presence of free fluid by a radiologist using a deep probe (2.5-3.5 MHz) of the Affiniti 50 ultrasound (Phillips, Netherlands). Presence of fluid in each of these spaces was considered as a positive result of ultrasound. Then, all patients underwent a diagnostic laparoscopy in the operating room under general anesthesia. For this purpose, a 10-mm trocar entered the abdomen 1 cm above the umbilicus. After insufflation using 5 liters of carbon dioxide gas, a laparoscopic camera was introduced through the trocar, and all the spaces inside the abdomen and both the diaphragms were examined with direct vision.

***Data gathering***

A questionnaire including information about age, gender, time to reach the emergency department, chest wall laceration length, chest wall laceration location, CT scan result, volume of chest tube fluid, and FAST and laparoscopy findings was filled for all enrolled patients. 

Research staff and Faculty were responsible for data gathering.

***Statistical analysis***

For reporting qualitative and quantitative data, frequency and percentages and mean ± standard deviation were used, respectively. Finally, the non-parametric Mann-Whitney U test was used to compare the means and the Fisher's exact test was used to compare the qualitative data in SPSS software version 21. P value of 0.05 or less was considered statistically significant.

## Results

***Baseline characteristics of the studied patients***

Twenty-four patients with the mean age of 33 ± 10.64 years (16-61 years) were studied (100% male). Table 1 shows baseline characteristics of the studied patients based on the presence or absence of diaphragmatic injury. The mean chest wall laceration size was 2.7 ± 2.7 cm (1-10 cm) and the most frequent location of wounds was posterior chest wall (42%) and in the 6^th^ and 7^th^intercostal space (50%). Chest tube was inserted for all patients, 11 (46%) patients had hemothorax, 7 (30%) patients had pneumothorax and 6 (24%) patients had hemopneumothorax. The average blood volume discharged from thorax at the time of chest tube insertion was 134 ± 125 cc.

***Diagnostic accuracy of ultrasonography***

All patients were evaluated by FAST and free fluid was present in the abdominal cavity of 2 (8.3%) patients. Diaphragmatic rupture was seen in 4 (16.7%) patients based on diagnostic laparoscopy, who underwent laparotomy for diaphragmatic rupture repair. In one of these patients, there was a simultaneous spleen laceration with a depth of 0.5 cm without active bleeding, which did not need splenectomy. In another patient, there was a laceration in seromuscular layer of stomach without obvious rupture or leak of content or signs of generalized peritonitis. In the other two patients, except for a diaphragmatic rupture, no other pathologic evidence was found in the abdomen. All patients were discharged from the hospital with good general condition and no mortality occurred. 

FAST had negative findings in all 20 patients whose diaphragm was intact, while the result of FAST was positive in 2 of 4 patients (50.0%) with diaphragmatic rupture. Based on this findings the sensitivity, specificity, positive and negative predictive values, and positive and negative likelihood ratio of FAST in detection of diaphragmatic raptures were 50% (95% CI: 9.18 – 90.8), 100% (95% CI: 79.9 – 100.0), 100% (95% CI: 19.8 – 100.0), 9.1% (95% CI: 1.5 – 30.6), Infinity, and 0.1 (95% CI: 0.02 – 0.37), respectively. The overall the accuracy of FAST in this regard (based on area under the ROC curve) was 75.0% (95% CI: 42.3 – 100.0; figure 1).

## Discussion

In this study, the prevalence of confirmed diaphragmatic rupture in patients with thoracoabdominal penetrating trauma was reported as 16.7%. The sensitivity and specificity of ultrasonography for diagnosis of diaphragmatic rupture in this study were 50% and 100%, respectively.

There was no significant association between mean age, time to reach emergency room, chest wall laceration size, and intercostal space number of penetrating trauma and the rupture of the diaphragm. However, diaphragmatic rupture was more probable in patients with more hemothorax or hemopneumothorax versus those with lower hemothorax volume or hemo/pneumothorax alone. Also, the diaphragm rupture rate was significantly higher in patients with penetrating trauma in the lateral chest wall compared to those in the anterior or posterior chest wall. 

In many studies, the rate of diaphragm rupture in patients with penetrating trauma of the left thoracoabdominal area is reported between 10% and 15% (9, 10). In a study by Yucel et al. in 2017 in Turkey, 81 patients referred to the emergency room with a penetrating trauma of the left thoracoabdominal area and underwent a diagnostic laparoscopy for detection of diaphragmatic rupture during a period of 6 years. Similar to our study, 92.6% of the patients were male and average age of the patients was 27.5 ± 9.8 years. Left diaphragm rupture was observed in 23.5% of patients. In this study, penetrating trauma to the chest wall was located at the anterior part in 55.5%, the lateral region in 33.3% and the posterior part in 11.2% of patients. Unlike our study, in this study, there was no association between the occurrence of penetrating chest trauma and presence or absence of hemopneumothorax with diaphragm rupture (11). In another study by Yucel et al. in Turkey in 2015, 43 patients with left thoracoabdominal trauma were examined for diaphragmatic rupture with CT scan and diagnostic laparoscopy in a 5-year interval. Similar to our study, 91% of patients were male and their average age was 30 years (15-60 years) and left diaphragm rupture was observed in 25.6% of the patients. 

Based on CT scan results, diaphragm was normal in 30 patients and diaphragmatic ruptures were diagnosed in 13. On the other hand, in diagnostic laparoscopy, diaphragmatic rupture was detected in 9 patients of 13 patients with positive CT scan and 2 of 30 patients with negative CT scan. In this study, sensitivity and specificity of CT scan in diagnosis of diaphragmatic rupture were 82% and 88%, respectively, and positive and negative predictive values ​​were 69% and 93%, respectively (12). In a study done by Bagheri et al. in Iran in 2009 on 30 patients with thoracoabdominal penetrating wound, 73.3% of the patients were male and their mean age was 26.2 years. Similar to our study, the most common location for trauma was in the 6th and 7th intercostal space. Similar to our results, 16.7% of patients had a diaphragmatic rupture, and diaphragm rupture was more frequently observed in those with wounds located between 7^th^ intercostal space and lower spaces (3). In a study by Mjoli et al. in 2009 in South Africa on 55 patients with penetrating thoracoabdominal trauma, 90% of the patients were male, with an average age of 26.3 years. Diaphragmatic rupture was seen in 40% of patients. The mean length of chest laceration was 3.1 ± 3.1 cm. The incidence of penetrating trauma to the chest was 41.8% in the lateral, 36.4% in the posterior and 20% in the anterior walls (13). The only study that examined the value of FAST in diaphragmatic rupture diagnosis was conducted in the United States in 2004 by Tayal et al. In this study, 8 patients with anterior penetrating thoracoabdominal trauma were evaluated using FAST and then underwent laparotomy based on the presence of intraperitoneal fluid in FAST evaluation. In contrast to our study, sensitivity of 100% (95% confidence interval: 63.1% -100%) and specificity of 100% (confidence intervals of 85.8% -100%) were reported for FAST in diaphragmatic rupture diagnosis (14). The difference of sensitivity of FAST between this study and our study can be due to the lower number of patients studied or selection of patients with penetrating trauma in the right or left side of the thoracoabdominal area in Tayal study. Due to the very proximity of the liver to the right diaphragm, the sharp object passing from the right diaphragm following a penetrating trauma to the right thoracoabdominal area definitively causes liver rupture and intra-abdominal hemorrhage, which is associated with positive FAST evaluation.

FAST in patients with penetrating trauma to the left thoracoabdominal cannot be the definitive alternative to diagnostic laparoscopy for detection of diaphragmatic rupture. Positive result of FAST in such patients suggests diaphragmatic rupture, so that these patients can undergo urgent laparoscopic surgery for diaphragm rupture repair without the need for laparoscopic examination. However, due to the small number of patients in the current study, more studies should be performed on a larger number of patients with penetrating chest trauma to reach a more precise decision about the role of FAST in diagnosis of diaphragm rupture.

## Limitations

One of our limitations was the relatively small sample size of the study. Larger studies with more patients might more precisely reveal the issues with using FAST in thoracoabdominal stab wound. However, it is suggested to follow-up patients for longer periods to check probable long-term complications. Another limitation of this study was operator dependency of FAST, which cannot be avoided. FAST performed by an expert radiologist might yield different results.

## Conclusion: 

Considering the low sensitivity of ultrasonography in detection of diaphragmatic rupture following penetrating trauma to the left thoracoabdominal region, it cannot be the definitive alternative to diagnostic laparoscopy for detection of diaphragmatic rupture.
